# Gender prediction based on University students’ complex thinking competency: An analysis from machine learning approaches

**DOI:** 10.1007/s10639-023-11831-4

**Published:** 2023-06-13

**Authors:** Gerardo Ibarra-Vazquez, María Soledad Ramí­rez-Montoya, Hugo Terashima

**Affiliations:** 1 Institute for the Future of Education, Tecnologico de Monterrey, Av. Eugenio Garza Sada 2501, Monterrey, 64849 Nuevo León Mexico; 2 School of Engineering and Sciences, Tecnologico de Monterrey, Av. Eugenio Garza Sada 2501, Monterrey, 64849 Nuevo León Mexico

**Keywords:** Complex thinking, Reasoning for complexity, Machine learning, Gender prediction, Higher education, Educational innovation

## Abstract

This article aims to study machine learning models to determine their performance in classifying students by gender based on their perception of complex thinking competency. Data were collected from a convenience sample of 605 students from a private university in Mexico with the eComplexity instrument. In this study, we consider the following data analyses: 1) predict students’ gender based on their perception of complex thinking competency and sub-competencies from a 25 items questionnaire, 2) analyze models’ performance during training and testing stages, and 3) study the models’ prediction bias through a confusion matrix analysis. Our results confirm the hypothesis that the four machine learning models (Random Forest, Support Vector Machines, Multi-layer Perception, and One-Dimensional Convolutional Neural Network) can find sufficient differences in the eComplexity data to classify correctly up to 96.94% and 82.14% of the students’ gender in the training and testing stage, respectively. The confusion matrix analysis revealed partiality in gender prediction among all machine learning models, even though we have applied an oversampling method to reduce the imbalance dataset. It showed that the most frequent error was to predict Male students as Female class. This paper provides empirical support for analyzing perception data through machine learning models in survey research. This work proposed a novel educational practice based on developing complex thinking competency and machine learning models to facilitate educational itineraries adapted to the training needs of each group to reduce social gaps existing due to gender.

## Introduction

The development of complex thinking encompasses high-level thinking and is considered as a competency encompassing different sub-competencies. By including cognitive and metacognitive processes, complex thinking can be postulated as a metacompetency (Silva Pacheco, 2020) that includes the sub-competencies of critical, systemic, and scientific thinking (Vázquez-Parra et al., 2022), plus innovative thinking (Ramírez-Montoya et al., 2022). It is essential to define the complexity approach used to advance knowledge, as there are fundamental epistemological, ontological and methodological differences (Sigahi and Sznelwar, 2022), and environmental knowledge and scientific literacy of students (Sholahuddin et al., 2021). For example, in healthcare, Mohammadi-Shahboulaghi et al. (2021) relate it to clinical reasoning identified as the cognitive process underlying clinical judgment, appropriate decision making, nursing quality improvement, metacognitive awareness, and professional competency in nursing. Cruzata-Martínez et al. (2022) promoted critical reading that is done collaboratively to develop higher cognitive skills by enabling complex thinking; expanding vocabulary and establishing strategies oriented toward shared emergencies. It is essential to point out that the mega-competency of complex thinking requires intention and action in the formative processes.

Promoting complex thinking implies integrating contextual components, active pedagogical intentions, innovative training processes, and interdisciplinary problem-solving. Morin (2007) states that all knowledge today needs reflection and should be recognized, situated and problematized. In citizen science, for example, it is crucial to promote projects with contextual awareness, citizen participation, infrastructure leveraging, technological innovation, educational innovation, dissemination and scale, networking and complex thinking (Sanabria-Z et al. , 2022). Also, Tuesca-Molina et al. (2021) found that incorporating the pandemic film genre, accompanied by the observation-relationship-application strategy, and using perceptions and beliefs questionnaire, improved the teaching-learning process, favored the approach to complex thinking problems, and improved empathy among teachers and students in the classroom. It is essential to understand that processes should not only address students; teachers also must strengthen their evaluative, critical and complex thinking skills (Belolutskaya et al. , 2022). Interdisciplinarity is a key factor in the formation of complex thinking (Baena-Rojas et al. , 2022). In this sense, Re (2020) indicates that complexity makes it possible to build new creative and critical curricula with multidisciplinary thinking, connecting fragmented knowledge, defending one’s own cultural identity based on the philosophy of care, and responding, in parallel, to the challenges of a politically imposed globalization. How could artificial intelligence help identify components and patterns in this framework and support predictions for complex thinking training processes?

It is essential to expand the analysis and instruments for complex thinking competency to equip individuals with the necessary skills to navigate the increasingly complex and dynamic world and effectively solve the multifaceted problems that arise. Conventional research methods in the educational field have already evaluated progress and shortcomings in this competency, considering sociodemographic factors (Vázquez-Parra et al. , 2022; Tobón and Luna-Nemecio , 2021). However, traditional research designs in educational sciences are at a turning point due to the expected effect of artificial intelligence. UNESCO (2019) and OECD (2021), leading international organizations, recommend incorporating artificial intelligence in educational research to improve the quality of educational systems. The classic model of linear regression and machine learning is based on the realization of predictive models of the data. However, machine learning, as a predictive statistical model with a robust non-parametric component, provides greater flexibility and a better classification of the different subgroups in the perception of the level of competency. This helps researchers and education experts create more personalized curricula adapted to the training needs that a specific group may require, optimizing teaching strategies and learning evolution to improve academic performance in complex thinking skills.

This article aims to study machine learning models to determine their performance in classifying students by gender based on their perception of complex thinking competency. The article is organized as follows: Section 2 presents the theoretical framework for the complex thinking competency and machine learning. In Section 3, we exhibit the associated research questions and the strategy pursued to experiment. Section 4 presents the experimental results, and in Section 5, we discuss contrasting the data with related works. To conclude with Section 6 where we highlight the findings and the study limitations, implications for practice and research, and suggestions for future studies.

## Theoretical background

### Complex thinking competency

Developing complex thinking is vital due to the social complexity and the challenge of educating for global and sustainable citizenship. This concept is based on the ability of people to connect different aspects of reality, not accepting facts as immovable truths but examining and comprehensively exploring them, using various forms of thought (de Melo , 2020). The field of education has been translated as a mega-competency that integrates four sub-competencies, according to Baena-Rojas et al. (2022): (1) scientific thinking deals with the ability to analyze problems and find explanations for natural and social phenomena through the scientific method; (2) systematic thinking is the ability to solve problems in a complex system, using an approach that considers the system as a whole and examines the interaction of its parts; (3) innovative thinking refers to the ability to propose solutions and answers beyond the ordinary or what conventional norms dictate, and (4) critical thinking involves analyzing and evaluating information about a specific topic, discovering the truth, and avoiding prejudice.

The processes involved in producing knowledge do not occur independently; instead, they are impacted by human social interaction, which can differ based on sociodemographic factors such as age, gender, and cultural heritage (Thiele et al., 2016). For this reason, in analyzing the per- caption of the level of competency in complex thinking, sociodemographic variables, such as gender, are considered to describe the population under study. This allows interpretations and predictions that invite introspection and search for adapted training to their characteristics (Tobón and Luna-Nemecio, 2021). Furthermore, incorporating gender in research reduces biases and improves the accuracy of training proposals linked to complex thinking sub-competencies: (1) It has been shown that one of the necessary elements to develop critical and scientific thinking sub-competencies is the genesis of the historical consciousness in which the lack of visibility of women in study plans or school curricula influences critical consciousness and scientific identity (Smyth and Nosek, 2015; 2) Gender differences have been found in the distribution of scores in the creative thinking sub-competency, specifically in domain-specific patterns in divergent thinking and creative problem-solving (He and Wong, 2021), and (3) The sub-competency of systemic thinking has been considered in the review and reformulation of educational actions that promote gender equality and the empowerment of women and girls as one of the Sustainable Development Goals (Acosta-Pasrischa, 2020). Therefore, considering the gender variable in the study of complex thinking mega-competency makes it possible to identify better and diagnose educational needs to promote effective changes in educational practices.

Educational research is committed to identifying and evaluating essential predictors of academic achievement. However, traditional educational research methods are based mainly on the positivist and phenomenological paradigms provide less precision than artificial intelligence techniques (Zhang and Aslan, 2021). Developing complex thinking requires reformulating simplistic pedagogical practices based on mechanical and rote learning (Sigahi et al., 2023). In this sense, machine learning is considered an emerging technology in the educational field that can respond to this challenge with greater rigor (Su et al., 2022). Despite this, it has yet to be explored so far in the scientific literature in this competition.

### Machine learning

Machine Learning (ML) is a sub-field of Artificial Intelligence (AI); it develops computer programs that learn and improve from experience without being explicitly programmed (Bishop, 2006). These computer programs utilize data to discover patterns to make predictions based on the given examples to perform a task. Contrary to traditional survey analysis methods, most ML computer programs do not require distributional data assumptions or explicit model specifications before estimation (Burkart and Huber, 2021). ML can broaden data processing to help survey researchers look at various aspects of perception studies. For example, Rojas-Córdova et al. (2020) employed predictive machine learning models to analyze the impact on Chilean companies’ innovation intention from perceived barriers. Using machine learning, Eder et al. (2021) studied fear and perceived health during the COVID-19 pandemic. Vowels et al. (2022) identified the most salient self-report predictors of perceived partner support cross-sectionally and six months later with a machine-learning model. However, an essential aspect concerning machine learning that usually is not considered is the bias in prediction. Mehrabi et al. (2021) reviewed different real-world applications that have shown biases and developed a taxonomy for fairness to avoid the existing bias in machine learning systems. For this reason, studying bias is essential when developing prediction models using perception data that reveal the partiality learned by the algorithm.

In education, machine learning models have been improving processes such as grading students, improving student retention, predicting student performance, and testing students. Manikandan and Chinnadurai (2020) explained that machine learning allows problem-solving in reasoning, knowledge representation, prediction, learning, and perception. Also, Korkmaz and Correia (2019) identified trends in machine learning in educational technologies, where they identified opportunities in using big data and learning analytics in education. Therefore, machine learning brings promising techniques for developing new educational models using students’ and teachers’ perceptions. For example, Suparwito et al. (2021) analyzed students’ perceptions of the online learning process to determine variables that influence the students’ satisfaction with online learning. Lin et al. (2021) applied machine learning to study the association between the length in the word count of a test item written in Chinese, item difficulty, and students’ perceptions about items in science term examinations. Salas-Rueda et al. (2022) analyzed the teachers’ perceptions about the school’s organization activities in Massive Open Online Courses (MOOCs) and Information and Communication Technologies (ICT) using machine learning models. Hew et al. (2020) employed machine learning to predict MOOC learner satisfaction and estimate their relative effects from specific learner-level and course-level factors.

## Methodology

Our aim is to study machine learning models to determine their performance in classifying students by gender based on their perception of complex thinking competency. We used perception data to addressed the following research questions:

- RQ1: Can machine learning build models to fit perception data, such as complex thinking?
- RQ2: Do machine learning models predict students’ gender using perception data of complex thinking employing new data to test?
- RQ3: Do machine learning models present bias in gender prediction?

### Participants

The data collected by Castillo-Martínez et al. (2022) came from convenience sample of $m=605$ students, of which 344 were females and 261 were males attending a private university in Mexico. The students belong to three different disciplinary areas: 1) Humanities, 2) Social Sciences, and 3) Engineering and Sciences. Table 1 shows the statistics about the students’ gender and disciplinary area. The data was collected through Google Forms with a self-assessment questionnaire answered voluntarily.

**Table 1:** Statistics from the students by gender

Disciplinary areas	Female	Male
Humanities	113	66
Social Science	122	55
Enginering and Science	109	140
Dataset	344	261

### Instrument

The eComplexity instrument designed by Castillo-Martínez et al. (2022) consisted of 25 items that measured the participants’ perception of their complex thinking competency and sub-competencies. The instrument is divided into four sub-competencies:

- Systemic thinking (items 1, 2, 3, 4, 5, 6, 7, 8);
- Scientific thinking (items 9, 10, 11, 12, 13, 14);
- Critical thinking (items 15, 16, 17, 18, 19, 20, 21);
- Innovative thinking (items 22, 23, 24, 25).

The instrument used a Likert scale (1: Strongly disagree, 2: Disagree, 3: Neither agree nor disagree, 4: Agree, 5: Strongly agree) to level the self-assessment questionnaire.

### Dataset preprocessing

Data preprocessing is crucial to finding optimal machine learning performances in the classification task. It comprises data integration, removing outliers, dealing with imbalanced data, and transforming data to common ranges (García et al. , 2016). For example, when constructing a dataset, some instances occur rarely or are difficult to sample. Therefore, such datasets occasionally encounter imbalanced data, which refers to the skewed class proportion in the data. For machine learning models, predicting the classes with a small proportion is, in the best case, a rare scenario because most of the time, small classes are undiscovered or ignored (He and Garcia , 2009). Then, the instances belonging to a small class are misclassified repeatedly to the classes of the majority proportion in the dataset. Most popular classification algorithms have a severe problem training with imbalanced class distribution datasets because they assume a relatively balanced distribution (Pulgar et al., 2017).

In binary classification problems, the ratio of the sample size of the small class to that of the majority class denotes the degree of imbalance distribution. Classification performance has also been associated with the class distribution from the dataset (Weiss and Provost , 2003). Better results were related to nearly balanced class distributions. However, sample size and class separability also degrade the classification model’s performance, making it almost impossible to state such deterioration to the imbalance distribution problem (Kumar et al. , 2021).

Resampling methods arise as a possible solution at the data level for imbalanced data, which refers to changing the distribution sizes of the classes. In binary problems, oversampling and downsampling find the most favorable class distribution by either increasing the small class size or decreasing the majority class size (Yap et al. , 2014). In oversampling, instances from the small class are randomly increased with duplicate samples to level the size to the majority class. In downsampling, instances from the majority class are randomly discarded to balance the size of the small class.

Data normalization is also an essential pre-processing data procedure to achieve optimal classification performance in machine learning models (Singh and Singh, 2020). Data normalization diminishes the bias of features with high numerical contributions in the learning process of machine learning models. So, features with high numerical values are transformed to a common range where they cannot influence the pattern recognition process more than features with small numerical values. All features in the data are equally contributing to the learning process. Therefore, to predict an instance’s class, all data features are equally important in the forecasting process (Pan et al., 2016). A standard normalization procedure for each feature from a questionnaire uses the following equation:

1 $$\begin{aligned} x_{normalized}=\frac{x-\mu }{\sigma } \end{aligned}$$

where $\mu$ is the mean, and $\sigma$ is the standard deviation of each item’s responses.

### Gender prediction

Machine learning can transform education by analyzing data to identify patterns and insights that inform educational decision-makers, personalize learning experiences, improve student outcomes, and enhance administrative tasks (Khan and Ghosh, 2021). This technology has the potential to revolutionize education and deliver personalized, compelling learning experiences to every student. Thus, an unexplored area is to analyze competency perception data to analyze students’ perceptions of education that considers features derived from data. This can be particularly valuable in identifying discriminant characteristics from data to enable educators to tailor their approaches to individual needs.

Model selection is a critical process in machine learning that involves choosing the most appropriate model for a given problem (Raschka, 2018). Selecting a suitable model is essential to achieving accurate and reliable predictions and insights derived from the data. Different models may have varying complexity, flexibility, and accuracy levels and may perform better or worse depending on the specific task. According to the literature, predictive machine learning models have been largely applied in Education Data Mining, which uses machine learning models to analyze large datasets to identify patterns and gain insights into student behavior, performance, and engagement (Abu Saa et al., 2019; Tomasevic et al., 2020; Kumar et al., 2018). From these literature reviews, we focus our work on three of the most found in these works: 1) Multi-layer perceptron, 2) Random forest, and 3) Support vector machines. Convolutional Neural Networks (CNNs) have recently become the standard in several Computer Vision and Machine Learning tasks (Li et al., 2021). Henceforth, the variant of 1D-CNNs was suggested and quickly demonstrated state-of-the-art performance in several research areas, such as categorizing personalized biomedical data and early diagnosis, structural health monitoring, and identifying anomalies and faults in power electronics and electrical motors (Kiranyaz et al., 2021). As a result, we consider applying 1D Convolutional neural networks to an education task. Therefore, we briefly describe each machine learning model and the theory behind them.

**Fig. 1:**
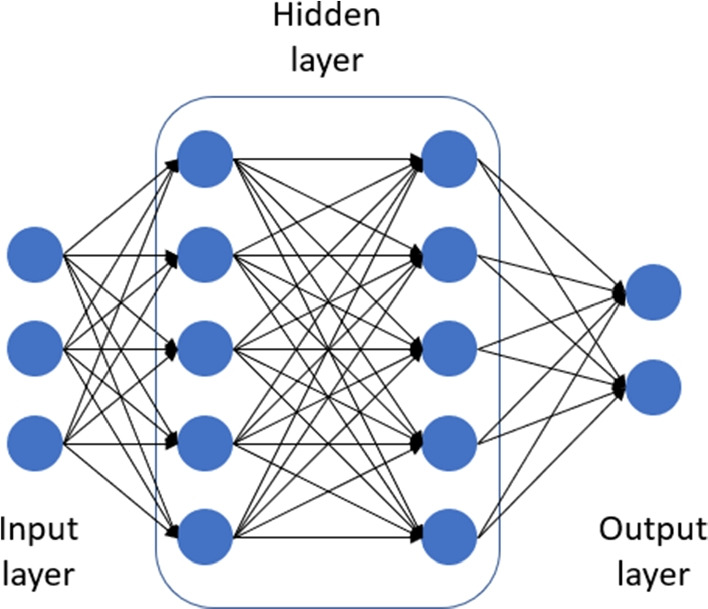
Layer’s structure of the Multi-Layer Perceptron

The *multi-layer perceptron* (MLP) is an artificial neural network model for classification tasks Hertz et al. (2018). Its architecture comprises three elements: an input layer, a hidden layer, and an output layer. Each layer is constructed with many perceptrons, which are the artificial model of neurons Rosenblatt (1958). A hidden layer is usually seen as a black box layer that allows putting inside more than one layer of perceptrons. Perceptrons from the hidden and output layers pose an activation function that permits the scale of the output and makes it differentiable to apply the backpropagation algorithm to train the model by iteratively adjusting its parameters so that the difference between the output and the target class is minimized. In Fig. 1, we illustrate an example of the layer’s structure of a Multi-Layer Perceptron.

**Fig. 2:**
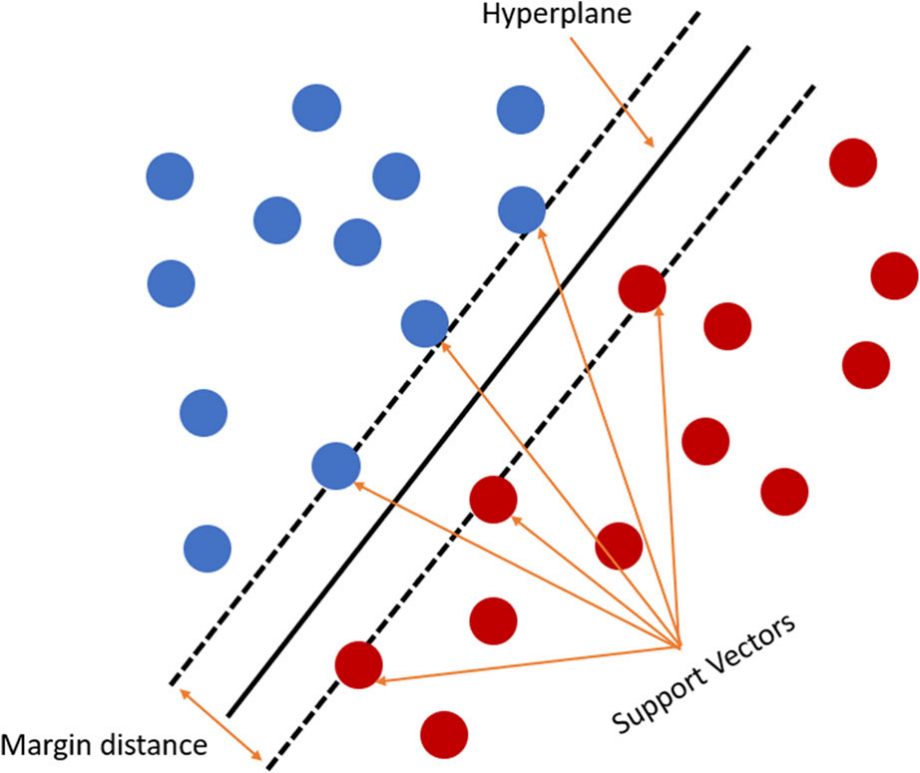
Basic example of the Support Vector Machines

One of the most used binary classification algorithms is the *Support Vector Machines* (SVM). It searches for an optimal separating hyperplane with the margin distance (Cortes and Vapnik , 1995). It means to find the minimal distance from the closest data points to the hyperplane, which separate the two classes. Data points close to the hyperplane are named as support vectors. Figure 2 shows a basic example of the hyperplane, the margin distance, and the support vectors from the SVM. Non-linear problems are also tackled by linear SVMs, in which the problem is mapped using non-linear basis functions in a high dimensional feature space. The feature mapping is obtained as a weighted sum of the values of a kernel function calculated at the support vectors (Noble , 2006). The class imbalance problem is supposed to affect SVMs less because the hyperplane is computed at the support vectors (selected data points), and the class size should not affect drastically compared to other classification algorithms (Japkowicz and Stephen , 2002).

**Fig. 3:**
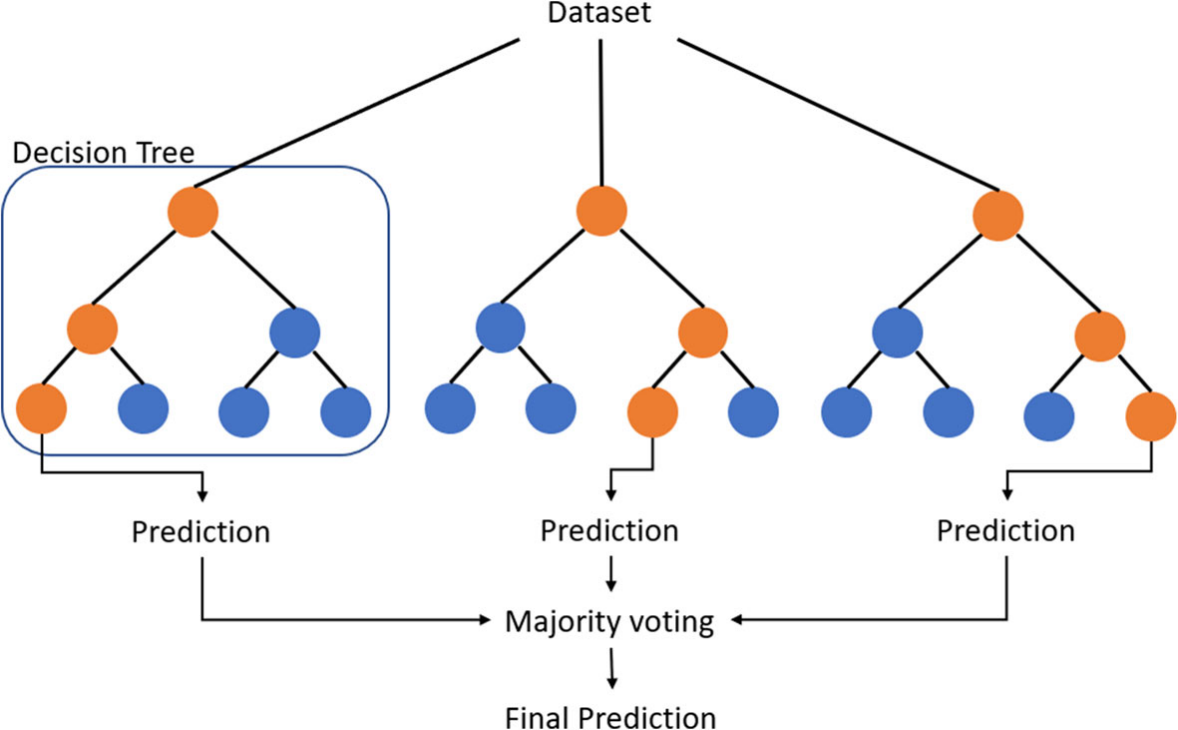
Example of random forest

*Random Forest* (RF) algorithms are popular machine learning algorithms for linear and non-linear data in classification and regression tasks (Breiman , 2001). These algorithms construct a series of decision trees using different samples and take the majority voting for assigning a class. Decision trees characteristically tend to create models that overfit the training data (Fawagreh et al. , 2014). However, Random Forest improves this behavior with a forest of decision trees that function to average the outcome of multiple trees trained on diverse sections of training data to reduce the variance. In Fig. 3, we outline an example of the structure of a random forest model to classify data.

**Fig. 4:**
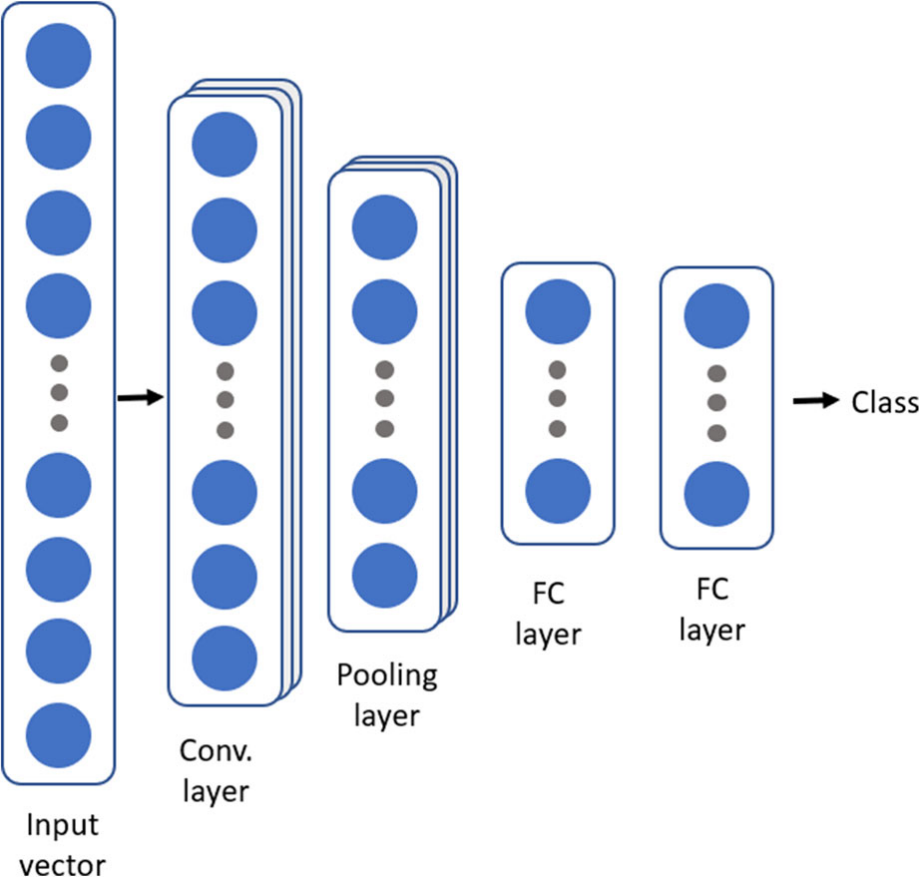
Example of convolutional neural network architecture

*Convolutional Neural Networks* (CNNs) have become the most popular Artificial Neural Networks for most computer vision tasks and machine learning operations. Their architecture is divided into multiple learning stages comprised of convolutional layers, activation functions, sub-sampling or pooling layers, and fully-connected layers, which are inspired by biological animal visual perception (Hubel and Wiesel , 1968). However, this technique has become the main instrument for 2D signals, such as images. Recently, CNNs for 1D signals have been proposed, and they have instantly become state-of-the-art in many 1D applications such as speech recognition, real-time electrocardiogram monitoring, and high power engine fault monitoring among others (Kiranyaz et al. , 2021). Figure 4 shows an example of the layers structure of a CNN for 1D applications.

To analyze each model’s performance we use *accuracy*, one of the most used metrics in classification. It simply calculates the rate of correct predictions given by the following formula:

2 $$\begin{aligned} Accuracy= \frac{1}{N}\sum _{n=1}^{N} d(y^{\prime }_{n},y_{n}) \;\;\;, \end{aligned}$$

where *N* is the total instances, $y^{\prime }_{n}$ is the predicted label, and $y_{n}$ is the original label for the instance *n*, and $d(y^{\prime },y)=1$ if $y^{\prime }=y$ and 0 otherwise. Nevertheless, accuracy can be misleading because a single number could not reveal the types of errors the model is making. A confusion matrix is a technique that visually presents the count relations between predictions (columns) and actual values (rows). It can give a better idea when the classification model explicitly confuses one class with another. To read binary confusion matrices correctly, the left diagonal shows the correctly classified instances while the right diagonal shows the incorrectly classified instances.

## Experiment and results

In this Section, we aim to outline the experimental procedures of our study and present the results obtained. The goal of this work is to analyze machine learning models performance in classifying students by gender based on their perception of complex thinking. To ensure that the data is accurate and suitable for the analysis, we conducted a thorough preprocessing stage. Table 2 shows the dataset configuration during the preprocessing procedure (data normalization and oversampling). Firstly, we use the standard data normalization from (1) for each item in the dataset. Then, we randomly oversampled the minority class (Male class) to balance the dataset by adding random duplicated instances from the minority class. We finished with 344 samples for each class, as shown in the *balanced data* column in Table 2. Then, we split the data to produce the training and testing datasets for the classification algorithms. Table 2 shows the instances for each class in each dataset. Training datasets are approximately 90% of the data, and testing datasets are approximately 10%.

**Table 2:** Dataset configuration

Gender	Original	Balanced data	Training	Testing
Female	344	344	318	26
Male	261	344	314	30

We use Scikit-learn v0.21.2 for building the MLP, RF, and SVM models and Keras v2.3.1 for the 1D-CNN implementation. Table 3 shows the layer configuration for the 1D-CNN. The experiments were run in a computer with Intel core i5-1145G7 with 16 GB of RAM, and we outline next the empirical tune selection of the hyperparameters for training each model:

- **MLP**: hidden layer size=[150,64,10], optimizer=*lbfgs*, alpha=$1 \times 10^{-5}$.
- **RF**: number of trees in the forest=*1000*, maximum depth of the tree=*15*.
- **SVM**:kernel=*radial basis functions*, tolerance for stopping criterion=$1 \times 10^{-3}$, decision function=*one versus one*.
- **1D-CNN**: loss function=*sparse categorical cross entropy*, optimizer=*Adamax*, metric=*sparse categorical accuracy*, batch size=*5*, epochs=*20*.

**Table 3:** 1D-CNN layers configuration

1D-CNN layers	
input ($25\times 1$)	
1D-Convolutional layer of 64 filters, and kernel size = 5	
Maxpooling layer of pool size = 5	
Fully Connected layer of 1000 neurons	
Fully Connected layer of 2 neurons	
soft-max layer	

Table 4 shows the results for each model at each stage of training and testing data. We show the corresponding computed accuracy from (2) at the end of each stage. We observed almost a similar behavior among all the classifiers in the training phase. Three of four classifiers (MLP, RF and 1D-CNN) obtained approximately 96% accuracy with the training instances. Contrarily, SVM obtained 79.75% of accuracy with the same dataset. In the testing stage, the classifier performances behave differently. The most accurate model was 1D-CNN with 82.14% of correct gender predictions of the testing data. Then, SVM obtained 80.36% of accuracy, RF obtained 76.79%, and MLP obtained 73.21%.

**Table 4:** Results for each model at each stage of training and testing

Algorithm	Training accuracy	Testing Accuracy
MLP	96.94%	73.21%
RF	96.36%	76.79%
SVM	79.75%	80.36%
1D-CNN	96.04%	82.14%

**Fig. 5:**
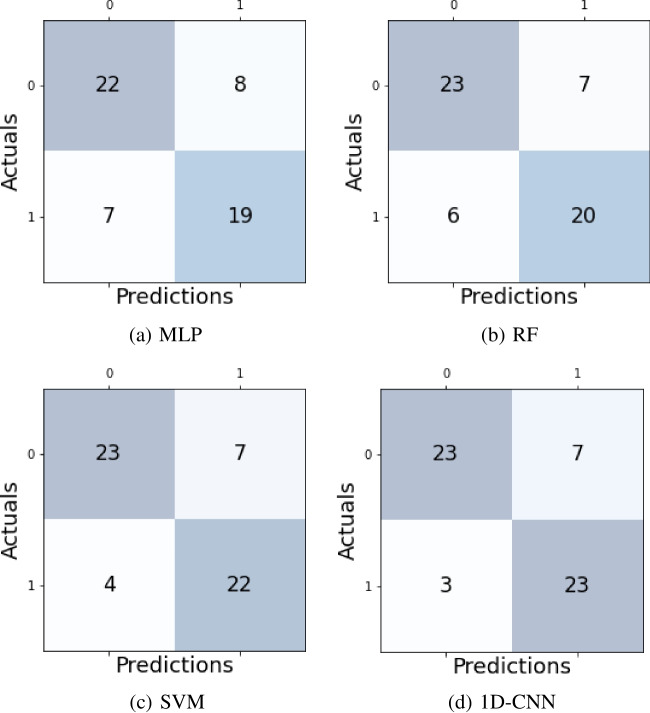
Confusion Matrices

Figure 5 shows the visualization of each model’s performance with the confusion matrix in the testing stage. In this sense, to clearly understand the confusion matrix, the upper left corner shows the correct prediction from the classifier for the *Male* class, and the bottom right corner shows the correct prediction for the *Female* class. The upper right corner shows the *Male* instances that the classifier predicted as *Female*, and the bottom left corner shows the *Female* instances that the model forecast as *Male*.

Figure 5a presents the MLP’s confusion matrix. It shows the number of instances where the model was inaccurate; eight students were classified as *Female*, but the students were *Male*. In the same direction, seven students were predicted as *Male*, but they were *Female*. For RF, the confusion matrix showed less error with seven students incorrectly classified as *Female* and six misclassified as *Male* as shown in Fig. 5b. For SVM and 1D-CNN, the confusion matrices present the same seven students falsely classified as *Female*, and the only difference is that 1D-CNN only has three inaccurately predicted *Males* against the four instances of SVM as shown in Fig. 5c, d.

## Discussion

The present study utilized machine learning models to predict gender based on students’ perceptions about their complex thinking competency as measured by a self-assessment questionnaire. Table 4 shows that the machine learning models effectively created mathematical models that accurately predict gender based on the training data. The models achieved an impressive accuracy rate of up to 96.94% for three out of four cases. It highlights the potential of machine learning in making accurate predictions based on self-reported data. Furthermore, the study contributes to the broader discussion on the use of machine learning in fitting mathematical models to predict various outcomes based on students’ attitudes and perceptions. This argument answered the first research question and agreed with the study’s findings and those of Suparwito et al. (2021) and Lin et al. (2021). Specifically, Suparwito et al. (2021) used a machine learning model (RF) to fit students’ perceptions of the online learning process. In contrast, Lin et al. (2021) used a machine learning model to associate the word count length of a test item written in Chinese, item difficulty, and student perceptions of the item. While the results of the present study are promising, it is essential to acknowledge the limitations and potential ethical implications associated with using machine learning in predicting a sensitive characteristic such as gender. As such, future studies should address these concerns by employing appropriate safeguards and ethical guidelines to avoid misuse, such as gender discrimination.

Machine learning models demonstrated to correctly predict new data and address RQ2 by achieving up to 82.14% accuracy with a novel state-of-the-art model in education 1D-CNN. Interestingly, Table 4 highlighted a difference in performance between the models in the testing stage, with SVM performing second-best at 80.36% accuracy, contrary to its training score. This finding suggests that models may perform differently in different stages and emphasizes the importance of testing models on new data. These results align with previous studies, such as Salas-Rueda et al. (2022), who employed predictive regression models to identify conditions of teachers’ perception of MOOCs and ICT with a squared error of up to 0.442 using testing instances. Hew et al. (2020) also predicted MOOC learner satisfaction and estimated their relative effects from specific learner-level and course-level factors, obtaining a mean F1 score of 0.822. Overall, the findings of the current study contribute to the growing body of literature on the use of machine learning models to make predictions based on students’ perceptions. The results demonstrate that while some models may perform well in the training stage, their behavior in the testing stage may vary, which implies the importance of selecting an appropriate machine learning model for a prediction based on students’ perceptions is crucial as it can significantly impact the accuracy of the predictions and the insights derived from the data. Furthermore, the study highlights the potential of machine learning models to make accurate gender predictions based on students’ perceptions about their complex thinking competency, providing a foundation for future research in this area.

The current study employed confusion matrix analysis to evaluate the gender prediction performance of all machine learning models and address the third research question. The findings revealed a partiality in gender prediction among all models, with the most frequent error being to predict *Male* students as *Female* class. The initial imbalanced data could explain this effect, suggesting that machine learning models may exhibit bias when trained on imbalanced datasets. Our results align with previous research by Mehrabi et al. (2021) and Navarro et al. (2021), who also examined algorithms’ unfairness and the risk of bias in supervised machine learning-based prediction models. These studies demonstrate the importance of considering potential biases in machine learning models, mainly when working with imbalanced datasets. However, oversampling was found to reduce the bias in the current study, resulting in high accuracy rates of up to 96.94% and 82.14% in the training and testing stages, respectively. These findings highlight the potential of oversampling techniques to improve the performance of machine learning models when working with imbalanced datasets. Altogether, the results of this study underscore the importance of addressing bias in machine learning models when making predictions based on student data. By identifying potential preferences and using oversampling techniques, machine learning models can achieve more accurate predictions, leading to more effective educational interventions and policy decisions.

## Conclusions

This article aims to study machine learning models to determine their performance in classifying students by gender based on their perception of complex thinking. The results confirmed our hypothesis that data from the eComplexity instrument provided sufficient information to build machine learning models to predict a student’s discriminant feature. In our study, we found: 1) machine learning models (Random Forest, Support Vector Machines, Decision Trees, and One-Dimensional Convolutional Neural Network) fit complex thinking competency perception data to forecast students’ gender, 2) the four machine learning models can find sufficient differences in the eComplexity data to classify correctly up to 96.94% and 82% of the students’ gender in the training and testing stage, respectively, and 3) confusion matrix analysis revealed partiality in gender prediction among all machine learning models, even though we have applied an oversampling method to reduce the imbalance dataset. Machine learning demonstrated the capability to build models that fit perception data and predicted students’ gender based on their perception of complex thinking competency. Therefore, mathematical models built by machine learning proved to learn patterns from human perception data and predict a student’s discriminant characteristic.

For survey researchers, machine learning models propose to expand the tools for data analysis, where traditional methods were too complicated to implement with the distributional assumptions and explicit model specification before prediction. Adaptive designs, data processing, and non-response adjustments are areas for survey researchers as machine learning models are becoming popular. Therefore, we conclude that machine learning models were efficiently implemented in our work to predict the students’ gender with a self-assessment questionnaire measuring the complex thinking competency. This paper provides empirical support for analyzing perception data through machine learning models in survey research. This work proposed a novel educational practice based on developing complex thinking competency and machine learning models to facilitate educational itineraries adapted to the training needs of each group to reduce social gaps existing due to gender.

Machine learning models are powerful and more flexible tools that learn from experience to perform some tasks like classification. However, one of the main problems of machine learning is that they are not easily interpretable by humans; they are seen as black-box models. Data is another limitation of machine learning models; a typical classification problem could demand a thousand to tens of thousands of instances to build a classification model. However, our results demonstrated that for our purpose, it was possible to build a model to predict students’ gender with up to 82.14% accuracy in the testing stage with a limited sample size of 605 students. We envision collecting new data to improve the models built by machine learning. This work points to the importance of machine learning techniques as tools for data analysis to build models to predict students’ gender with a self-assessment questionnaire of complex thinking competency. This research proposes to continue exploring machine learning applications and models in perception surveys to predict important features from data that can provide algorithms to understand human perceptions to be integrated in new educational models.

## Data Availability

The datasets generated during and/or analysed during the current study are available from the corresponding author on reasonable request.
